# Assessing Nasal Epithelial Dynamics: Impact of the Natural Nasal Cycle on Intranasal Spray Deposition

**DOI:** 10.3390/ph17010073

**Published:** 2024-01-06

**Authors:** Amr Seifelnasr, Xiuhua Si, Jinxiang Xi

**Affiliations:** 1 Department of Biomedical Engineering, University of Massachusetts, Lowell, MA 01854, USA; amr_seifelnasr@student.uml.edu; 2 Department of Mechanical Engineering, California Baptist University, Riverside, CA 92504, USA; asi@calbaptist.edu

**Keywords:** intranasal spray deposition, nasal cycle, turbinate, deposition patterns, targeted drug delivery, nasal morphology, nasal congestion, decongestion, nasal resistance

## Abstract

This study investigated the intricate dynamics of intranasal spray deposition within nasal models, considering variations in head orientation and stages of the nasal cycle. Employing controlled delivery conditions, we compared the deposition patterns of saline nasal sprays in models representing congestion (N1), normal (N0), and decongestion (P1, P2) during one nasal cycle. The results highlighted the impact of the nasal cycle on spray distribution, with congestion leading to confined deposition and decongestion allowing for broader dispersion of spray droplets and increased sedimentation towards the posterior turbinate. In particular, the progressive nasal dilation from N1 to P2 decreased the spray deposition in the middle turbinate. The head angle, in conjunction with the nasal cycle, significantly influenced the nasal spray deposition distribution, affecting targeted drug delivery within the nasal cavity. Despite controlled parameters, a notable variance in deposition was observed, emphasizing the complex interplay of gravity, flow shear, nasal cycle, and nasal morphology. The magnitude of variance increased as the head tilt angle increased backward from upright to 22.5° to 45° due to increasing gravity and liquid film destabilization, especially under decongestion conditions (P1, P2). This study’s findings underscore the importance of considering both natural physiological variations and head orientation in optimizing intranasal drug delivery.

## 1. Introduction

The nasal cycle is a phenomenon in which the majority of airflow initially passes through one side and then the other side of the nose, depending on the side experiencing the most resistance to airflow. In approximately 80% of adults, this cyclic variation in nasal resistance has a frequency ranging from 50 min to 4 h [1]. The nasal cycle is driven by an imbalance in blood flow, causing swelling of erectile cells in the nasal septum and inferior turbinate of one passage compared to the other. This uneven enlargement of tissue obstructs the airflow more in one passage, which ventilates different amounts of inhaled chemicals to the olfactory mucosa, facilitating the localization of the odor source [2]. Vasodilation on one side occurs simultaneously with vasoconstriction on the opposite side, causing the predominant airflow to shift between nasal passages while the overall resistance remains constant. During periods of increased respiratory activity, such as exercise or deep breathing, nasal resistance decreases, likely due to vessels becoming decongested [1].

The purpose of the nasal cycle is a subject of discussion. Some research suggests that the alternating nasal airflows function as a means for conditioning air, eliminating trapped pollutants, facilitating mucociliary clearance, and localizing the odor origin [3,4]. Conversely, other theories propose that the nasal cycle plays a role in safeguarding against respiratory infections, or it serves as an indicator of these physiological states [5,6,7]. The exact mechanisms of how the circadian rhythm governs the nasal cycle remain unclear. However, it is believed that the autonomic nervous system plays a vital part in this process. The occurrence of nasal cycle can vary with age, physical activity, and specific medication [8,9,10,11,12]. Furthermore, factors that cause the widening of nasal blood vessels, thickening of the nasal mucosa, and alterations in nasal secretion levels, like allergic, nonallergic, or infectious forms of rhinitis, also affect the nasal cycle’s occurrence [13,14].

Intranasal drug administration is firmly established for treating nasal diseases and is gaining recognition as an effective alternative to oral and injectable methods. The nasal mucosa has increasingly been identified as a feasible pathway for systemic drug delivery [15]. The structure and function of the nasal cavity offer distinct benefits for reaching targets in both the respiratory and olfactory regions, facilitating targeted or systemic drug delivery. The efficacy of intranasal drugs relies on a combination of factors related to the drug, delivery device, and the patient. These encompass characteristics of the drug formulation, design of the delivery device, administration method, target of deposition, and health conditions of the nose [16,17]. The variability in nasal anatomy among individuals is especially significant in influencing intranasal drug delivery, as it has a significant impact on the drug distribution in the nose and, thus, the effectiveness of the drug.

Nasal anatomy and morphology impact the effectiveness of intranasal drug delivery and can be significantly affected by nasal diseases, nasal obstruction, and the periodic turbinate congestion/decongestion from the natural nasal cycle. Nasal obstruction, which limits airflow, can be caused by various pathologies, including the common cold, rhinosinusitis, nasal polyps, nonallergic rhinitis, and allergic rhinitis [18]. These factors pose challenges in achieving consistent and optimal drug absorption through the nasal mucosa, particularly when targeting specific regions in the nose for therapeutic effects. For instance, the effectiveness of nasal irrigation can be influenced by the nasal cycle, which alters nasal geometry and resistance [19].

Many intranasally administered medications, such as nasal sprays, rely on inspiratory flow to enhance their effectiveness in reaching target regions within the nasal cavity. Inspiratory flow helps facilitate the movement of the medication through the nasal passages and promotes optimal drug delivery to the desired areas for absorption or a therapeutic effect. Nasal obstruction or congestion can thus increase nasal airflow resistance, potentially hindering the optimal intranasal delivery to target regions. Measuring nasal airflow directly in living organisms is difficult, leading to the frequent use of computational fluid dynamics (CFD) and experimental techniques like particle image velocimetry (PIV) to study respiratory airflows [20,21,22,23,24]. The findings of several studies illustrate the impact of anatomical structures within the nasal cavity on nasal resistance [25,26,27,28,29]. Bickford et al. indicated that decongestants can notably decrease the resistance of the nasal airway [30]. Xi et al. investigated the nasal dilation effects on olfactory deposition and found that nasal dilation could double the delivery efficiency to the olfactory region [31]. However, there is limited quantification of the influence of the nasal cycle on nasal airflows and the dosimetry of intranasal drug delivery. Eccles et al. reported that the nasal cycle could induce a nearly fourfold alteration in the resistance of a unilateral nasal passage, suggesting a significant impact on the behaviors and fates of aerosolized drugs delivered intranasally [32].

According to a study by Xiao et al., nasal decongestants lead to a notable expansion in the passage posterior to the nasal valve by reducing the volume of the anterior turbinate [20]. Findings from their study suggest that, apart from reducing nasal cavity resistance by approximately 50%, decongestion also reduced the intersubject variability in nasal resistance across the test cohort. One noteworthy point is that the majority of patient-specific experimental or CFD studies utilize nasal cavity geometries acquired through medical imaging, capturing the airway at a single moment in time [33,34,35]. The objective of this study was to employ in vitro techniques to observe and investigate the impact of congestion/decongestion resulting from the natural nasal cycle on the penetration and distribution of administered nasal sprays within the nasal cavity. Specific aims included:

(1) Prepare a set of multi-sectioned transparent 3D-printed nasal cast models based on a patient-specific nasal geometry, incorporating varying degrees of turbinate thickness to reflect different levels of congestion/decongestion resulting from the nasal cycle.
(2) Visualize the distribution of deposited intranasally administered sprays within the various sections of each nasal model under the influence of inhalation flow and various head positions.
(3) Quantify the deposition in the various model sections: front nose, turbinate regions (anterior, middle, posterior), and nasopharynx, and evaluate the outcomes of the experimental scenarios.

The outcomes of this study will enhance our knowledge of how congestion and decongestion, which are part of the natural nasal cycle, affect the dosimetry of nasal sprays and the delivery of accurate doses to specific intranasal targets.

## 2. Results

### 2.1. Nasal Cycle Models and 3D-Printed Casts

**Figure 1:**
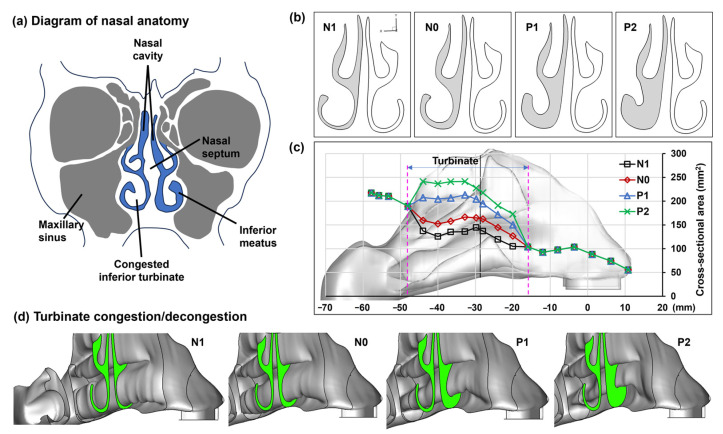
Variation in the left inferior meatus due to concha (turbinate) congestion/decongestion (N1–P2) within one nasal cycle: (**a**) diagram of nasal anatomy, (**b**) visualization of a selected cross section among N1–P2, (**c**) quantification of the cross-sectional area variation among N1–P2 in the turbinate region, and (**d**) turbinate morphological variation among N1–P2. N1: the left inferior meatus shrinks due to concha congestion; N0: normal; P1 and P2: the left inferior meatus dilates due to increasing concha decongestion.

Four nasal models were developed to represent nasal passage variations at different stages of the nasal cycle (Figure 1a). Here, N1 represents the passage shrinkage (i.e., negative) due to congestion and N0 represents the normal, while P1 and P2 represent the passage dilation (i.e., positive) due to increasing concha decongestions (Figure 1b). Figure 1c shows the morphological dimensions of the four models (N1, N0, P1, and P2). Because the congestion/decongestion occurred only on the conchae, the front nose and nasopharynx remained the same throughout the nasal cycle. To illustrate the three-dimensional structural change of the left inferior concha, the right side of the nose and the nasal septum were removed (see Figure 1d), revealing the gradual transition in the left inferior concha from a congested state (N1) to a normal condition (N0), and finally to decongested states (P1 and P2).

**Figure 2:**
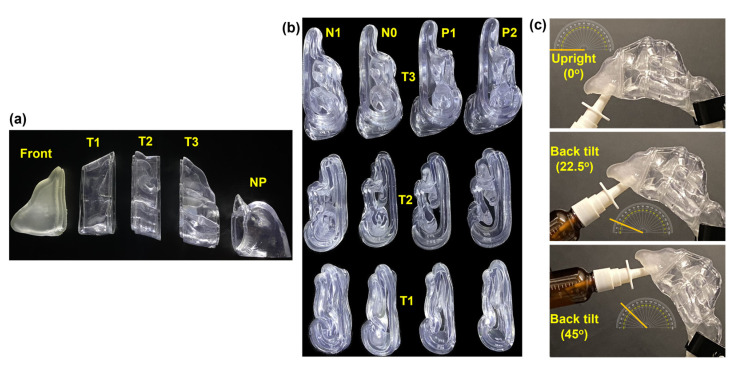
Nasal casts and experimental setup: (**a**) Nasal cast sections (front nose, T1, T2, T3, and nasopharynx [NP]) for deposition quantification. (**b**) Sectional views of the turbinate region sections of the four nasal casts (N1, N0, P1, and P2) illustrating varying inferior turbinate thickness. (**c**) Experimental setup depicting three head positions: upright, 22.5°, and 45° back tilts.

To quantify spray deposition distributions, each nasal model was divided into five sections, namely, the front nose, anterior turbinate (T1), middle turbinate (T2), posterior turbinate (T3), and nasopharynx (NP), as illustrated in Figure 2a. Changes in the morphology of the four nasal models were reflected in sections T1, T2, and T3 (Figure 2b). Lap joints were generated at the connecting ends to facilitate robust assembly and secure sealing. The assembled nasal casts (N1, N0, P1, and P2) were subsequently used in spray deposition tests with three head angles, i.e., upright (0°), 22.5° back tilt, and 45° back tilt (Figure 2c).

### 2.2. Spray Deposition vs. Nasal Cycle at a 22.5° Backward Head Tilt

#### 2.2.1. Time-Sequence Visualization of Deposition in Model N1

**Figure 3:**
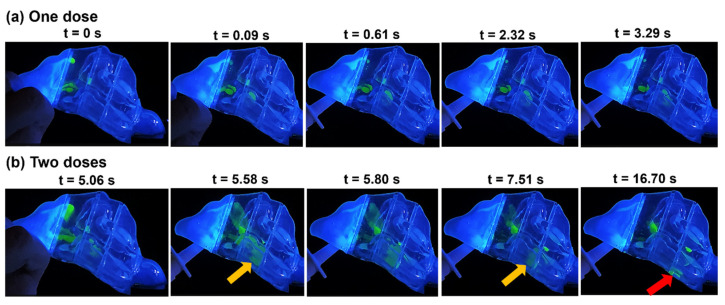
Time series visualization of droplet distribution, liquid film development, and translocation in the N1 model with a 22.5° backward tilt following: (**a**) a single spray pump dose and (**b**) two doses of spray applications.

Figure 3 depicts the time-sequence visualization of the liquid droplet and film deposition of intranasally administered saline nasal spray within the N1 nasal model, representing the most congested stage in a nasal cycle after one- and two-dose applications at a head orientation of 22.5° tilted backward.

Upon application of the initial dose at t = 0 s, a portion of the merged spray droplets settled in the superior front nose, and an almost equal volume was dragged by flow shear and gravity through the mid-anterior section of the turbinate region, depositing along the septum wall as well as the outer surface of the inferior turbinate anteriorly within sections T1 and T2 (at t = 0.09 s). Between 0.09 s and 3.29 s, the deposited film in section T1 translocated downwards and posteriorly, settling within the same section. In T2, the film spread wider downwards and, at t = 3.29 s, settled mostly in the narrow clearance between the inferior turbinate and septum wall.

After the second application at t = 5.06 s (Figure 3b), more doses were deposited in the roof of the front nose, adding to the liquid film that was deposited there after the first spray application. Furthermore, additional liquid film passed into T1 and merged with previously deposited liquid film in that section. Accumulated liquid film in the superior front nose was dragged by flow shear through the anterior section of the roof of the respiratory region, spreading across the septum wall and dragged further downwards and diagonally through T1 by gravity, passing the anterior and middle turbinate, and ultimately merging between t = 5.06 s and 5.58 s with film deposited inferiorly in T1 (Figure 3b). The film continued to spread and translocate diagonally under the combined effects of flow shear and gravity, only to extend within the very narrow cavity in T2 between the inferior turbinate and the septum (indicated by the orange arrow at t = 5.58 s). A large section of that liquid film detached at t = 5.80 s (Figure 3b), by which time inhalation flow had ceased, and translocated posteriorly through T2, maintaining its degree of spread across the very narrow passageway until reaching the very end of T2 (white arrow at t = 7.51 s, Figure 3b) and continued mobilizing into T3. Upon entering the T3 section of the turbinate region, the sectional area of the passage widened, causing the film to split into separate parts along the lining of the inferior turbinate and septum. With the lack of inspiratory airflow by the time the film entered T3, flow shear effects vanished. The vertical component of gravity was strong enough to overcome the liquid-surface adhesive force, detaching and splitting the liquid film and ultimately leading to its settlement on the nasal cavity floor in T3 (red arrow).

#### 2.2.2. Comparison of Spray Deposition Patterns among N1, N0, P1, and P2

**Figure 4:**
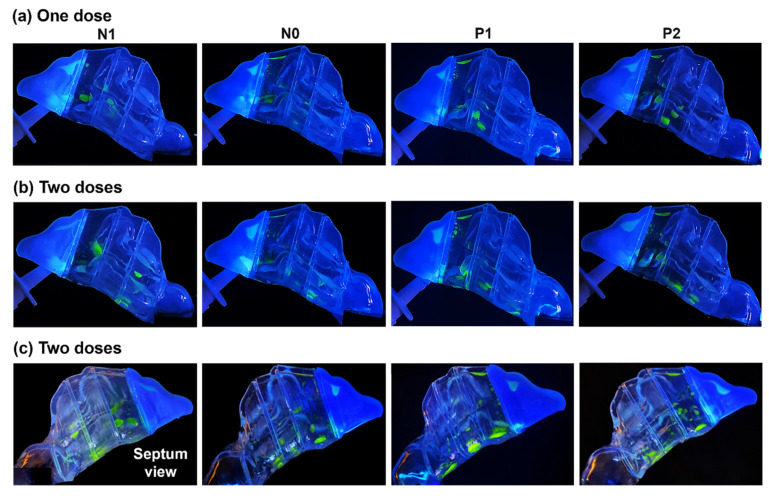
Liquid drop and film deposition within the various models with a 22.5° backward head tilt: (**a**) after one dose of spray application, (**b**) after two doses, and (**c**) after two doses captured from the septum side of each model.

The spray deposition distribution after one- and two-dose applications within the four models (N1, N0, P1, and P2), representing various degrees of congestion/decongestion throughout one nasal cycle, can be seen in Figure 4.

The first two rows depict the results of one- and two-dose applications, respectively, viewed from the lateral left side. The third row shows the captures from the nasal septum side. At the backward head tilt of 22.5°, in all models, a single-dose application led to a scatter of spray droplets and liquid film throughout the middle as well as the inferior regions of the T1 and T2 turbinate region sections, with some deposition in the front nose and the roof of anterior T1. P1 and P2 exhibited more deposition near or on the floor of T2 compared to models N1 and N0, where the deposition in that section was in the middle region between the outer surface of the inferior turbinate and septum. This is attributed to the wider inferior passages in P1 and P2, allowing for the wider spread of the spray droplets and sedimentation further towards the floor of T2. In contrast, the narrower passageway in the T2 section of N0, and even more so in N1, resulted in more liquid film settling in the tight space between the lining of the inferior turbinate and septum.

Upon administering a second dose, in addition to increased deposition in the front nose and slight additions to the superior region of T1, larger liquid film formations developed. Flow shear and gravity effects dragged more liquid posteriorly within the nasal cavity, resulting in deposition in the rearmost T3 section. It was observed that the narrower inferior passageways in N1 and N0 induced greater flow shear effects compared to P1 and P2, owing to higher flow velocities in those passages than in P1 and P2. When viewed from the septum side, a larger deposition distribution on or near the floor of the nasal cavity was evident in all sections of the turbinate region (T1, T2, and T3) of models P1 and P2 compared to N1 and N0. This is primarily attributed to the wider inferior passages of P1 and P2, as explained above.

### 2.3. Spray Deposition vs. Nasal Cycle at Upright and 45° Backward Head Tilt

**Figure 5:**
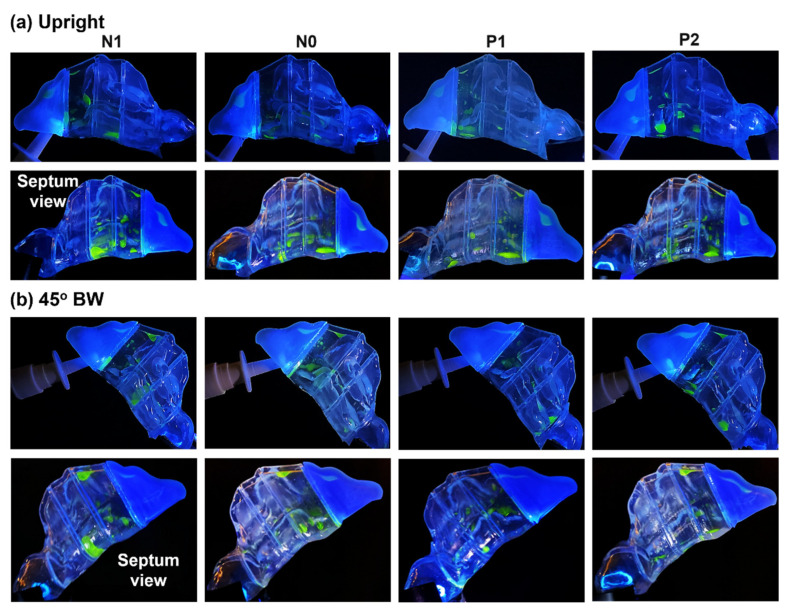
Liquid drop and film deposition within the various models after two doses of spray application with an (**a**) upright (0°) head angle and (**b**) 45° backward head tilt. The upper and lower panels in (**a**,**b**) are views from the lateral and septum side, respectively.

Figure 5 showcases the final deposition distribution after the application of two nasal spray doses in all four models (N1, N0, P1, and P2) at both an upright head orientation and a backward head tilt of 45°.

In an upright head position, liquid deposition in the turbinate region was mostly confined to the T1 and T2 sections, as well as at the interface between T2 and T3. Deposition in these regions resulted from the combined effects of inertial impaction, gravitational sedimentation, wall film formation, and flow shear drag. However, the effects of gravity, coupled with the convoluted morphology of the nasal cavity within the T1 and T2 sections in all the models, facilitated the settling of liquid droplets and film in the middle and inferior turbinate regions, with the majority of deposition close to or at the cavity floor. The narrower clearance between the outer surface of the inferior turbinate and the nasal septum in models N1 and N0 resulted in a wider scattering of spray droplets and film, both within and across this confined space. The dispersion extended from the middle zone of sections T1 and T2 downwards towards the nasal cavity floor. This is clearly depicted in the nasal septum view in the second row of Figure 5.

At a 45° back tilt head orientation, the steeper angle accentuated the gravitational component in the direction of the flow, which assisted flow shear in dragging administered spray droplets and film formations deeper into the entire nasal cavity, mostly through the middle meatus. This resulted in deposition distributions dispersed throughout all three turbinate region sections (T1, T2, T3, and T4) in all four models (N1, N0, P1, and P2). Moreover, compared to the upright head position, there was a larger film formation along the superior cavity of section T1, covering the entire length of this section. It is also worth mentioning that the seemingly large deposition in section T2 in the N1 model, as seen from its septum side, is actually due to the spread of liquid film formation within and across the very narrow passage clearance between the congested inferior turbinate’s outer lining and that of the nasal septum wall.

### 2.4. Quantification of Regional Deposition vs. Nasal Cycle and Head Angle

**Figure 6:**
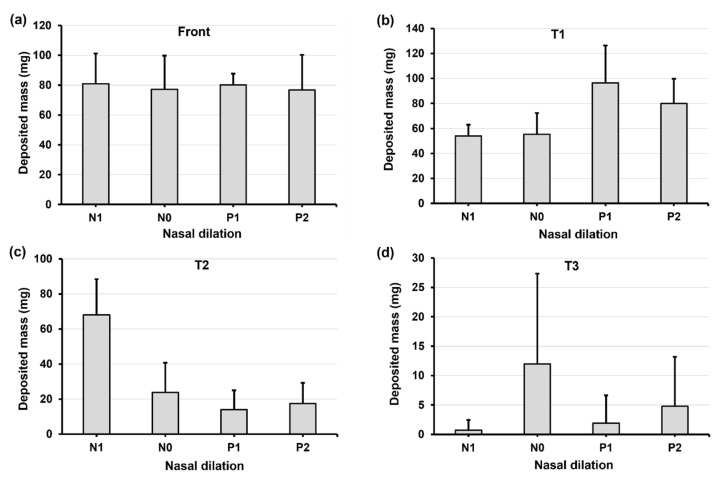
Deposition variation vs. nasal cycle (N1, N0, P1, and P2) in different regions of the nose with an upright head position: (**a**) front nose, (**b**) T1, (**c**) T2, and (**d**) T3.

Figure 6 shows the deposition variation vs. nasal cycle (N1, N0, P1, and P2) in different regions of the nose (front nose, T1, T2, and T3) with an upright (0°) head position. Insignificant differences in deposition among N1–P2 are observed in the front nose (Figure 6a), which is expected because the nasal cycle affects only the turbinate region, not the front nose.

Considering the deposition in the turbinate, the deposition in the middle turbinate region section (T2) gradually decreases from N1 to P2 (Figure 6c), while the trend appears to be irregular in both T1 (anterior turbinate section, Figure 6b) and T3 (posterior turbinate section, Figure 6d). This irregularity is more pronounced in T3, with much higher deposition in model N0 than in the other three models (Figure 6d). Large variances in deposition are also observed in T3, indicating a high level of liquid film instability in this region. It is noted that the spray deposition depends on both the initial deposition and subsequent translocation of the deposited liquid films, which are sensitive to gravity and local anatomical topologies. The gradual turbinate decongestion from N1 to P2 has at least three effects on the spray deposition: (1) the turbinate shrinks, reducing the surface area for droplet deposition and liquid film attachment; (2) the inferior meatus widens, allowing the spray plume to penetrate deeper; and (3) the turbinate topology changes, affecting the stability of the deposited liquid film. Thus, the gradual decrease in T2 deposition may result from the decreased turbinate area. In T1 deposition, the gradual increase from N1 to P1 may result from the widening meatus, while the slight decrease from P1 to P2 can be attributed to the shrinking turbinate. It is also noted that interactions exist among the local deposition mass, local turbinate topology, and associated gravity, leading to a nonlinear deposition distribution in T1, T2, and T3. In this test case, the abnormally higher T3 deposition in model N0 is a direct result of its drastic deposition decreases in T2 (Figure 6d vs. Figure 6c).

**Figure 7:**
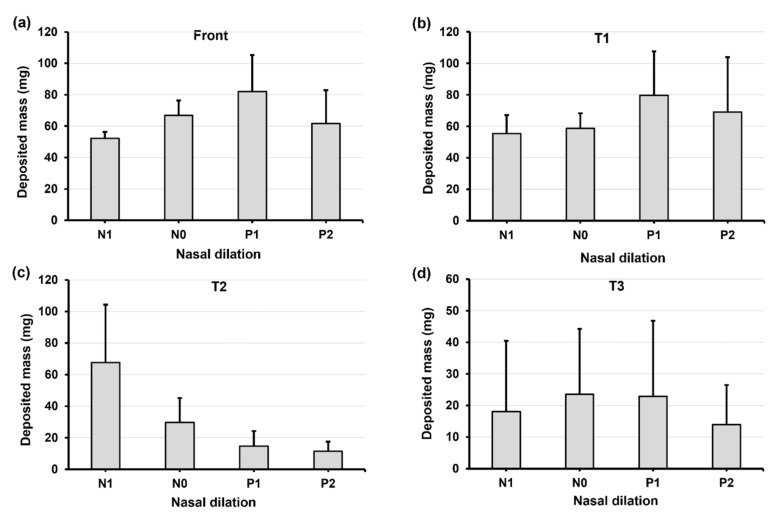
Deposition variation vs. nasal cycle (N1, N0, P1, and P2) in different regions of the nose with a 22.5° back tilt head position: (**a**) front nose, (**b**) T1, (**c**) T2, and (**d**) T3.

**Figure 8:**
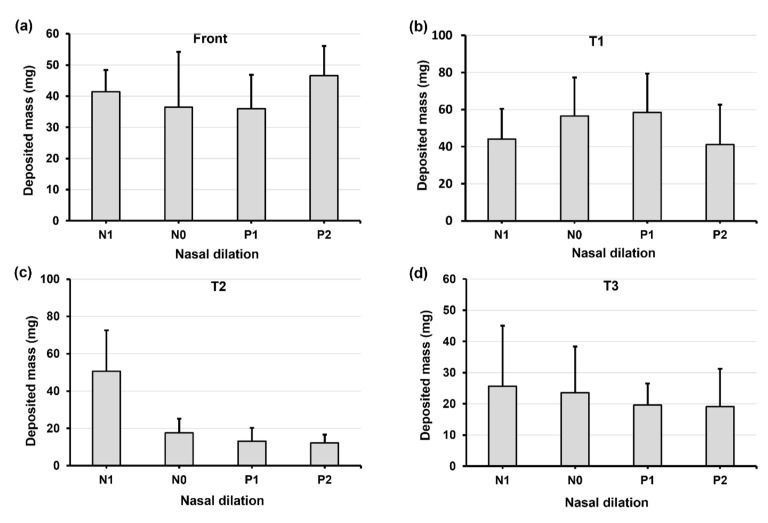
Deposition variation vs. nasal cycle (N1, N0, P1, and P2) in different regions of the nose with a 45° back tilt head position: (**a**) front nose, (**b**) T1, (**c**) T2, and (**d**) T3.

Figure 7 and Figure 8 show the deposition variation vs. nasal cycle (N1, N0, P1, and P2) with a head position back tilt of 22.5° and 45°, respectively. Together with Figure 6 with an upright head position (i.e., 0°), it is observed that the head angle exerts a significant effect on the spray deposition distribution and noticeably regulates the influence of the nasal cycle. In Figure 7 with a 22.5° back tilt head angle, the front nose dose peaks in model P1 (Figure 7a). Within a nasal cycle with a decongesting turbinate (N1–P2), the regional dose persistently increases in T1 while it decreases in T2 (Figure 7b,c). Again, large dose variances are noted in T3, reaffirming the high level of instability of the liquid film in upstream regions.

When increasing the head angle to 45° (Figure 8), we observe different patterns in deposition distribution among N1–P2 compared to those at 0° and 22.5°, i.e., with a relatively flat pattern in the front nose (Figure 8a), a modal pattern in T1 (Figure 8b), and a constant decrease pattern in T2 (Figure 8c) and T3 (Figure 8d). These patterns can be attributed to the predominant gravitational effect at 45°, which drives the deposited liquid film downstream of the nasal passage and, at the same time, leaves a thinner liquid film attaching onto the turbinate wall. This competing effect explains the dose increase from N1 to P1 (gravity-driven film translocation) and decrease from P1 to P2 (decreasing turbinate area). In T2 and T3 (Figure 8c,d), the constant decrease from N1 to P2 comes from the progressively shrinking turbinate. It is also observed that certain doses exit the nasopharynx in models P1 and P2 due to the pronounced gravity-driven translation at 45°.

### 2.5. Factorial Analyses of Effects of Nasal Cycle and Head Angle

#### 2.5.1. Total Deposition

**Figure 9:**
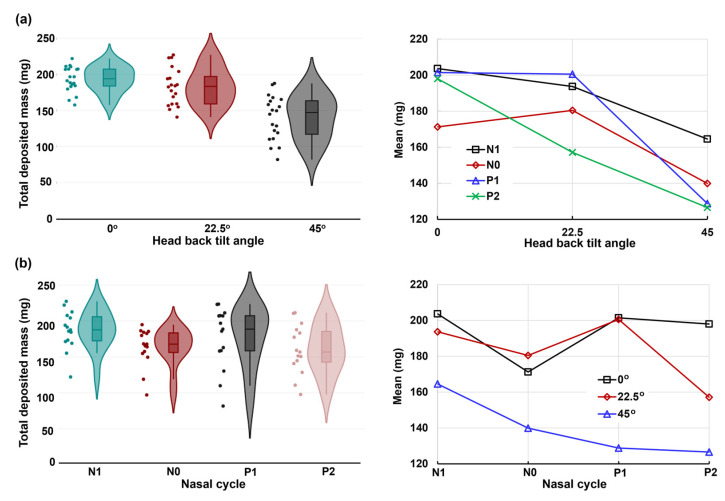
The violin and mean plots of the total deposited mass in four nasal models (N1–P2) and three head orientations: (**a**) head angle effect and (**b**) nasal dilation effect.

Figure 9 displays both the violin and mean plots illustrating the total deposition concerning head angle and nasal dilation due to the nasal cycle. The statistical analysis of total deposition, considering both factors (head angle and nasal cycle), is prominently featured in Figure 9. The violin plot further highlights the variances in total deposited mass concerning head angle (Figure 9a) and nasal cycle (Figure 9b), revealing substantial deposition variations in both factors. Significant deposition variances are evident in both factors, with the head angle exerting a more pronounced impact relative to the nasal cycle effect. This is evidenced by a noticeable decrease in deposition from the upright nose position (0°) to a 22.5° back tilt, followed by a drastic decrease from 22.5° to a 45° back tilt. Moreover, the magnitude of variance escalates as the back tilt angle increases, causing a diverse range of movements of the liquid film within the nasal cavity.

The mean plots in the right panels of Figure 9a,b provide insight into the two-factor ANOVA concerning head angle and nasal cycle effects along the *x*-axis, respectively. The corresponding *p*-values are listed in Table 1. As expected, a highly significant difference (*p*-value < 0.001) emerges in total deposition across the three head angles (0°, 22.5°, and 45°). Additionally, a very significant difference (*p*-value = 0.003) surfaces among the four models, reflecting nasal cycle-induced turbinate congestion/decongestion (N1, N0, P1, and P2). Notably, the interaction between the head angle and nasal cycle effect significantly influences total deposition, as indicated by a *p*-value of 0.031.

#### 2.5.2. Regional Deposition

**Figure 10:**
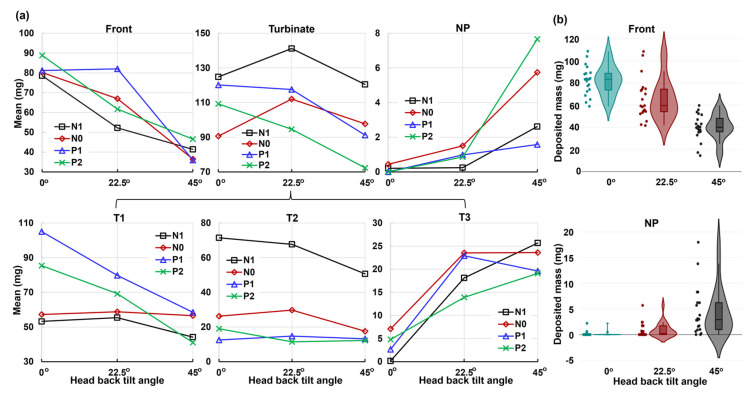
Factorial analyses vs. head angle for regional deposition in four nasal models (N1–P2): (**a**) mean plot and (**b**) violin plot.

**Figure 11:**
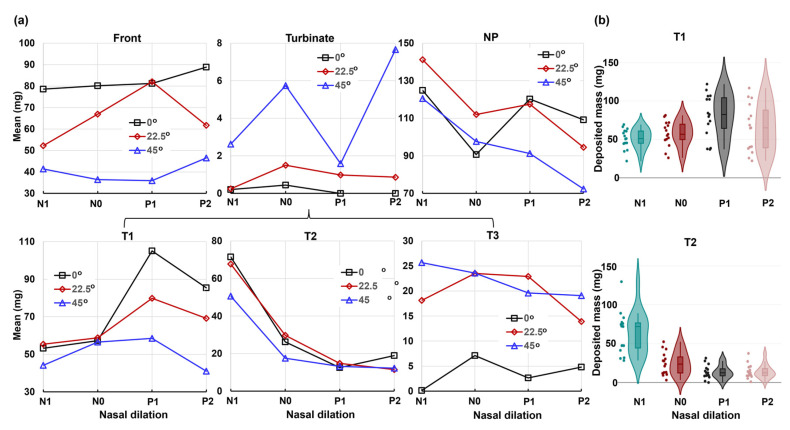
Factorial analyses vs. nasal cycle for regional deposition with three head orientations: (**a**) mean plot vs. nasal dilation and (**b**) violin plot.

Figure 10 displays the mean and violin plots for regional deposition concerning the head angle on the *x*-axis, while Figure 11 represents the nasal dilation on the *x*-axis.

The regions considered include the front nose, turbinate, and nasopharynx (NP), with the turbinate further divided into T1, T2, and T3 (Figure 10a and Figure 11a). The corresponding *p*-values for individual regions are listed in Table 1. The analysis reveals that the head angle significantly influences deposition in the front nose, T1, T3, and NP, with highly significant *p*-values (*p*-value ≤ 0.001). This influence is visually illustrated in the violin plots for the front nose and NP in Figure 10b. However, the head angle’s effect on turbinate deposition is significant (*p*-value = 0.034) but insignificant on T2 (middle turbinate), which is consistent with spray reaching the middle turbinate in all test cases post-dose applications.

Conversely, the nasal cycle has a highly significant effect (*p*-value ≤ 0.001) on deposition in both the turbinate and the T2 section, as illustrated in the lower panel of Figure 11b. The nasal cycle effect is also very significant (*p*-value = 0.003) for spray deposition in T1 (anterior turbinate), as shown in Table 1 and the upper panel of Figure 11b. However, the nasal cycle effect is not significant for spray deposition in the front nose (*p*-value = 0.223), T3 (posterior turbinate, *p*-value = 0.78), and NP (*p*-value = 0.061). These results align with the expectation that the congestion/decongestion induced by the nasal cycle primarily affects the turbinate region, leaving the front and back of the nose largely unaffected by passage morphology. Considering the mutual effect between the head angle and nasal cycle, insignificant interactions are observed in all individual regions considered, with *p*-values exceeding 0.05 (Table 1). This contrasts with the significant interaction between the two factors for the entire region (Figure 9). The observed difference is speculated to be due to the overall slight decrease in regional deposition with increasing head angles (i.e., increasing gravitational effect), cumulatively resulting in a significant difference in total deposition.

## 3. Discussion

This study investigated the effects of the nasal cycle, in conjunction with varying head angles, on the deposition patterns of intranasally administered nasal sprays. The results highlight several key findings that contribute to our understanding of the complex dynamics involved.

### 3.1. Effects of Nasal Cycle on Spray Deposition Distribution

The nasal cycle, characterized by cyclic changes in nasal airflow resistance, plays a crucial role in shaping the deposition patterns of intranasal sprays. This study delves into the intricate relationship between the nasal cycle and nasal spray deposition, utilizing models representing stages of congestion and decongestion (N1, N0, P1, and P2). Consistent parameters such as spray formulation, pump nozzle angle, insertion depth, and inspiratory flow rate were maintained. Despite this, significant variations in deposition were observed across nasal cycle stages. During congestion (N1), the increased resistance in one passage led to a more confined deposition space in the narrow clearance between the inferior turbinate and septum. Decongestion (P1, P2) widened the passage, allowing for broader dispersion of spray droplets and increased sedimentation towards the floor of turbinate sections T1, T2, and T3 (congested N1 model in the first column vs. decongested P2 in the second column, Figure 4 and Figure 5).

### 3.2. Effects of Head Angle and Interactions with Nasal Cycle

Head orientation, combined with the nasal cycle, played a crucial role in spray distribution. In an upright position, deposition was confined to specific turbinate sections, namely, T1 and T2 within the congested N1 and normal N0 model, due to inertial impaction and gravitational sedimentation (first and second columns, Figure 5a). Flow shear effects were small in drawing fluid deeper into the narrow nasal passages. However, a 22.5° back tilt increased gravitational effects, aiding flow shear in dragging droplets deeper into the nasal cavity. A 45° tilt accentuated these effects and facilitated deeper penetration of spray droplets, predominantly through the middle meatus, dispersing spray throughout all the turbinate sections, T1, T2, and T3 (Figure 5b). The impact of head angle on deposition was substantial, and its interaction with the nasal cycle significantly influenced the overall spray distribution. Both factors contributed to the complexity of the deposition pattern observed in this study.

### 3.3. Deposition Variance Related to Nasal Cycle and Head Angle

Despite efforts to maintain controlled conditions, a notable variance in deposition was observed, as illustrated by the charts of deposition mass distributions in the four nasal models (N1–P2) with the various head positions (upright, 22.5°, and 45° back tilt head positions, Figure 6, Figure 7 and Figure 8). Figure 9, depicting the violin and mean plots of the total deposited mass throughout the various stages of congestion and decongestion, shows that the magnitude of variance increased as the head tilt angle increased backward from upright to 22.5° to 45°. This change occurred due to the increasing influence of gravity, leading to the destabilization of the liquid film and inducing a variety of movements in the liquid layer. This variability persisted across both nasal cycle stages and different head angles. Gravitational sedimentation, inertial impaction, flow shear, and the complex nasal cavity morphology contributed to this observed variability. The nonlinear nature of the variance suggests a multi-faceted interplay of factors. The nasal cycle, head angle, and convoluted nasal cavity geometry collectively contribute to the intricate and variable nature of the spray deposition within the nasal cavity.

### 3.4. Implications for Intranasal Spray Administration

The intricate interplay between the natural nasal cycle and the distribution patterns of intranasal sprays has significant implications for targeted drug delivery within the nasal cavity. The nasal cycle, marked by cyclic variations in nasal airflow resistance, exerts a substantial influence on intranasal spray distribution. Despite maintaining consistent parameters, the models representing congestion and decongestion stages (N1, N0, P1, and P2) display notable variations in deposition. During congestion (N1), elevated nasal resistance confines spray deposition, while decongestion (P1, P2) widens the nasal passage, leading to broader dispersion of spray droplets, especially towards the posterior turbinate section (T3) and nasopharynx. These regions are the target for mucosal immunization against respiratory infectious diseases, as well as the initial site of viral infections such as COVID-19 [36,37,38,39,40,41].

Furthermore, head orientation, in conjunction with the nasal cycle, proves to be a critical factor in spray distribution. In an upright position, deposition is confined to specific turbinate sections (T1 and T2), whereas back tilts (22.5° and 45°) increase gravitational effects, facilitating deeper droplet penetration. A 45° tilt notably enhances dispersion throughout turbinate sections T1, T2, and T3. The interaction of the head angle with the nasal cycle significantly influences overall spray distribution, impacting the targeted delivery to specific nasal regions.

### 3.5. Limitations

This study is subject to several limitations that should be considered in the interpretation of results. Firstly, the focus on morphological variation was confined to the inferior concha, specifically affecting the left inferior meatus. This choice simplifies the investigation, neglecting the cyclic changes in congestion and decongestion typically involving all turbinate regions (inferior, middle, and superior) within one nasal passage. Secondly, the study’s scope is limited by the relatively small variety of nasal models used. Although variations in the left inferior turbinate were explored across different phases of the nasal cycle, a more extensive range of patient-specific nasal models reflecting diverse nasal passage morphologies would provide a more accurate assessment of intersubject variability.

Additionally, the connection interfaces between different sections of the model utilized tongue-and-groove connections that were not watertight. In some instances, this design allowed the deposited saline liquid to seep through tight spaces at the connections via capillary action, leading to minor inaccuracies in deposition quantifications. Although these discrepancies were deemed insignificant, they should be acknowledged. Furthermore, this study utilized nasal cavity models constructed with SLA resin through 3D printing. This material choice differs from the in vivo compliant airway [42]. A life-condition nasal cavity comprises squamous epithelial tissue in the anterior (front) region and nasal mucosa in the turbinate region. A comprehensive evaluation of the liquid-surface properties of the nasal mucosa compared to the resin surface is warranted to understand the degree of similarity and potential implications for this study’s findings.

## 4. Materials and Methods

### 4.1. Study Design

To assess the impact of mucosal congestion and decongestion during the natural nasal cycle on intranasal spray deposition, four multi-piece anatomically accurate nasal models were developed, each with distinct turbinate profiles (N1, N0, P1, and P2; Figure 1b,c). To accurately assess the effects of turbinate congestion/decongestion resulting from the nasal cycle, it was necessary to incorporate factors facilitating the penetration of administered nasal sprays deep into the nasal passage. This approach allowed for a more comprehensive evaluation of the impact of nasal passage clearance along the entire length of the nasal cavity. Key influencing factors included the nasal spray delivery device, liquid formulation, administration angle, device nozzle insertion depth, number of spray applications (doses), head orientation, and inhalation flow rate. The nasal delivery device used was a refillable multi-dose nasal spray pump (Hengni) loaded with a saline solution comprising 0.65% *w/v* sodium chloride, benzalkonium chloride, monosodium phosphate, phenylcarbinol, and disodium phosphate. The rationale for using a saline solution in this study stems from its status as one of the most used formulations for nasal sprays, employed for purposes such as nasal irrigation, moisturizing, and clearing nasal passages.

The nozzle of the nasal spray pump was fixed at an angle of 30° from the nostril’s normal, pointing toward the internal nasal valve in all the experimental runs, and inserted at a fixed depth of approximately 10 mm. Two applications were administered for each test run, with a single application delivering an average dose of 95 mg of saline solution. Three different head orientations were considered to assess the influence of gravity on the spray deposition distribution in the various regions of the nasal cavity at various positions of the nasal cavity under the effects of the nasal morphology due to turbinate congestion and decongestion. Among the many factors affecting intranasal deposition, gravitational sedimentation can contribute to the deposition of larger droplets. The translocation paths of liquid film, developed from the merging of multiple droplets within the nasal cavity, are also susceptible to gravitational effects, which vary based on head position. Thus, the experimental runs utilized the following head orientations: upright, 22.5°, and 45° back tilts from the horizontal (Figure 2). These orientations aimed to evaluate the influence of gravity on spray distribution within various nasal cavity regions, corresponding to different head positions affected by nasal morphology changes due to mucosal congestion and decongestion. To generate inhalation flow, each model was connected to a vacuum via a flexible tube (Robinair 3 CFM, Warren, MI, USA). The flow rate was set at a constant 16 L/min, corresponding to deep inhalation, only during nasal spray applications for all test cases and was regulated using a flow meter (Omega, FL-510, Stamford, CT, USA).

### 4.2. Nasal Cast Models

The nasal model N0, representing a normal condition, was previously developed from MRI scans of a 53-year-old male with no reported respiratory diseases [43,44]. The remaining three models (N1, P1, and P2) were modified from model N0, with changes made to the left inferior turbinate to reflect various degrees of congestion and decongestion. Thus, the four nasal models had a gradually widening nasal passage from a congested state (N1) to a normal condition (N0) and finally to decongested states (P1 and P2) throughout a nasal cycle, as illustrated in Figure 1. The geometrical modification was implemented using SolidWorks (Dassault Systems, Waltham, MA, USA).

The nasal casts were printed using a Formlabs 3D printer (Form 3B+, Somerville, MA, USA). The printing material was a clear stereolithography (SLA) rigid resin (Formlabs Clear Resin, FLGPCL04), creating transparent nasal casts (Figure 2). In contrast, the front nose section was printed using elastic SLA resin (Formlabs Elastic 50A Resin, FLELCL01) using the same 3D printer. This choice was made to simulate the flexible nature of the real front nose and allow for dilation, enabling the nasal spray pump’s nozzle to be inserted deeper into the nostril to achieve the desired insertion depth. A major benefit of employing transparent casts is the capability to visually monitor the real-time movement of liquid sprays administered intranasally and the resulting film formation within the nasal cast.

The rationale behind creating the nasal casts with only a single passage and an incorporated nasal septum was to facilitate clear observation and visualization of the spray deposition and liquid film translocation through the passage. This included views not only from the cheek side but also from the septum side, enabling visualization from the opposite side of the turbinate. This aspect would have been obscured if a complete nasal model had been used.

### 4.3. Experimental Protocol

In conducting experiments for each head orientation, the sections (Front, T1, T2, T3, and NP) of each nasal model (N1, N0, P1, and P2) were assembled using clear adhesive tape wrapped around the outside surfaces, securely and firmly fastening the parts together. The assembled models were then positioned to correspond to one of the three head orientations outlined in the study design, namely, upright, 22.5° back tilt, and 45° back tilt. After being filled with saline solution and properly primed, the nasal spray pump was fixed into place to achieve the desired administration angle and insertion depth. For each experimental run, the vacuum, connected to the model’s nasopharynx (NP) via a flexible tube, was activated for a short duration, simulating a period of deep inhalation. During this phase, two spray doses were administered successively, with a brief pause of a few seconds between applications.

A fluorescent green dye (GLO Effex, Murrieta, CA, USA) was used to visualize the dynamics of the liquid spray and subsequent liquid film translocation. A camera facing the side of the nasal model captured real-time intranasal spray dynamics, as well as the final deposition distribution on the nasal walls. An LED light with wavelengths ranging from 385 nm to 395 nm was used to illuminate the fluorescent dye. The deposited mass of liquid spray in various sections (Front, T1, T2, T3, and NP) was quantified for each model by measuring the mass of each section before and after each experimental run. This was accomplished with a Bonvoisin electronic scale with a precision of 0.1 mg (San Jose, CA, USA). A minimum of five repetitions were conducted for each experimental scenario to account for variability.

### 4.4. Statistical Analysis

The statistical analysis was performed using Minitab software 21.4 (State College, PA, USA). To assess result variability, a one-way analysis of variance (ANOVA) was conducted. The doses delivered in each test case were expressed as a range and presented as mean ± standard deviation. Deposition data statistics were illustrated using violin plots, which are akin to box plots and display the data’s probability density at various values, smoothed by a kernel density estimator [45]. ANOVA mean effect analysis was employed to evaluate the comparative influence of the nasal cycle and head angle. Statistical significance was determined when a *p*-value was less than 0.05, while a *p*-value ≤ 0.01 indicated a very significant difference and a *p*-value ≤ 0.001 denoted a highly significant difference.

## 5. Conclusions

In conclusion, this comprehensive investigation into intranasal spray deposition sheds light on critical factors influencing targeted drug delivery within the nasal cavity. This study underscores the intricate relationship between the natural nasal cycle, head orientation, and spray distribution patterns. Notably, congestion and decongestion stages induce significant variations in deposition, emphasizing the relevance of considering physiological conditions for optimal drug delivery. The impact of head angle, particularly in back tilts, is substantial, influencing gravitational effects and spray dispersion. The observed variance in deposition, even under controlled conditions, reflects the complex interplay of gravity, nasal cycle, flow shear, and cavity morphology. These findings provide valuable insights for enhancing targeted drug delivery strategies and emphasize the necessity of accounting for individual variability in nasal physiology.

## Figures and Tables

**Table 1:** The *p*-values from two-factor analyses of variance vs. the head angle and nasal dilation.

Region	Total	Front	Turbinate	NP	T1	T2	T3
**Head angle**	<0.001	<0.001	0.034	0.001	0.001	0.244	0.001
**Nasal dilation**	0.003	0.223	0.001	0.061	0.003	<0.001	0.78
**Interaction**	0.031	0.074	0.364	0.075	0.164	0.794	0.941

## Data Availability

Data is contained within the article.
